# Identifying long-term psychological distress from single measures: evidence from a nationally representative longitudinal survey of the Australian population

**DOI:** 10.1186/s12874-020-00938-8

**Published:** 2020-03-05

**Authors:** J. Welsh, R. J. Korda, E. Banks, L. Strazdins, G. Joshy, P. Butterworth

**Affiliations:** 1 Research School of Population Health, Australian National University, Building 62, Mills Rd, Acton, ACT 2601 Australia; 2 The Sax Institute, Ultimo, Australia; 3 Melbourne Institute of Applied Economic and Social Research, University of Melbourne, Melbourne, Australia

**Keywords:** Psychological distress, Kessler10, Measurement, Longitudinal, Statistical agreement

## Abstract

**Background:**

Single time-point assessments of psychological distress are often used to indicate chronic mental health problems, but the validity of this approach is unclear. The aims of this study were to investigate how a single assessment of distress relates to longer-term assessment and quantify misclassification from using single measures to indicate chronic distress.

**Methods:**

Data came from the Household, Income and Labour Dynamics in Australia Survey, a nationally representative study of Australian adults. Psychological distress, measured with the Kessler10 and categorised into low (scores:10- < 12), mild (12- < 16), moderate (16- < 22) and high (22–50), has been assessed in the Survey biennially since wave 7. Among respondents who were aged ≥25 years and participated in all waves in which distress was measured, we describe agreement in distress categories, and using a mixed linear model adjusting for age and sex we estimate change in scores, over a two-, four-, six- and eight-year follow-up period. We applied weights, benchmarked to the Australian population, to all analyses.

**Results:**

Two-years following initial assessment, proportions within identical categories of distress were 66.0% for low, 54.5% for mild, 44.0% for moderate and 50.3% for high, while 94.1% of those with low distress initially had low/mild distress and 81.4% with high distress initially had moderate/high distress. These patterns did not change materially as follow-up time increased. Over the full eight-year period, 77.3% of individuals with high distress initially reported high distress on ≥1 follow-up occasion. Age-and sex- adjusted change in K10 scores over a two-year period was 1.1, 0.5, − 0.7 and − 4.9 for low, mild, moderate and high distress, respectively, and also did not change materially as follow-up time increased.

**Conclusion:**

In the absence of repeated measures, single assessments are useful proxies for chronic distress. Our estimates could be used in bias analyses to quantify the magnitude of the bias resulting from use of single assessments to indicate chronic distress.

## Background

Depression and anxiety are the leading causes of morbidity in Australia [1], affecting 15 and 26% of the population at some point in their lives, respectively [2]. In addition to the often substantial negative effects they have on the mental, social and economic wellbeing of those affected, symptoms of depression and anxiety are also associated with chronic physical diseases – including cardiovascular disease [3] cancer [4], and dementia [5] – and all-cause mortality [6]. In order to investigate the associations between common mental disorders and physical health, measures of depression and anxiety are regularly included in cohort studies and used to predict a range of health outcomes over time.

Given that diagnosis using gold standard criteria and time-consuming structured diagnostic instruments is often not feasible in large-scale studies, brief mental health assessment tools are used on questionnaires to screen participants for likely mental disorders. Tools that are regularly used include the General Health Questionnaire [7], the Centre for Epidemiologic Studies Depression Scale [8] and the Depression, Anxiety and Stress Scales [9]. However, one of the most commonly used assessment tools is the Kessler 10 (K10), which was purposively designed to identify individuals with common mental disorders in community-based surveys [10]. The K10 assesses how often in the past 4 weeks respondents’ experienced ten non-specific symptoms of psychological distress, such as feeling “worthless”, “depressed” and “nervous”. Validation studies have shown that K10 scores are associated with symptoms of mental disorders, the associated disability and mental health care usage, and can be used to indicate the probability that the individual meets diagnostic criteria for disorder [11–13]. More than 85% of the Australian population with the highest K10 scores meet DSM-IV criteria for a current common mental disorder (i.e. any mood or anxiety disorder) or substance use disorder compared to approximately 5% of those with the lowest scores [11]. The combined brevity and validity has given the K10 broad appeal and it is now included in a number of prominent Australian and international health surveys, including the Australian Health Survey, the New Zealand Health Survey, the Canadian Community Survey and large scale linkage studies such as the 45 and Up Study.

While validation studies have demonstrated the utility of brief assessment tools to identify a *current* disorder, it is common to use single measures of distress as a proxy for chronic mental health problems. For example, single assessments of distress are used to predict health outcomes over long follow-up periods, often years or even decades after initial assessment (see for example: [6, 14, 15]). These studies imply that assessment of distress at a single time point can be used to identify more chronic problems because an isolated four-week period of distress is itself unlikely to be of importance for future health. However, given that depression and anxiety are generally episodic disorders, with a natural course, average duration and anticipated recurrence rate, levels of psychological distress may change considerably within individuals over time. Yet, the extent to which a single assessment of distress can be used to identify those who experience symptoms over the longer term is not well understood, despite its importance for understanding how distress influences physical illness. If large proportions of respondents are misclassified when using single assessments to indicate longer-term distress, there is potential for associations between distress and chronic physical diseases to be considerably biased.

Existing longitudinal research examining within-person change in broad symptoms of psychological distress measured with brief assessment tools is relatively uncommon [16] and the findings that are available are somewhat inconsistent. One study, using strict cut points to define categories of high compared to low distress, reported that of the 8.6% of men living in Australia aged 15 years and over who reported poor mental health at baseline, 61.5% were not categorised as having poor mental health 1 year later and for 92.4% their initial poor mental health had resolved within 9 years, suggesting that most people do not experience high levels of distress for long periods of time (findings were similar for women) [17]. In contrast, others have reported levels of distress are generally stable over time. One study found that within-person change in distress scores was small, with stability coefficients derived from structural equation models as high as 0.8 over a 10 year period [18]. Another, describing average change in depressive symptoms within a community-dwelling population aged 45 years and over, reported that although depressive symptoms increased slightly as people aged, symptoms were relatively stable over at least 13 years [19]. The differences in these findings may, at least in part, reflect the different methodologies used. Using strict cut points potentially overestimates variability in symptoms of distress by missing a proportion of the population who fall below cut points but who continue to experience symptoms. However, quantifying average change across the population may underestimate variability by masking large positive and negative changes within the same population, as well as differences at opposing ends of the distress scale. Given that the majority of the population reports no or only low levels of symptoms of distress and that many mental disorders are episodic, it may be the case that lower levels of psychological distress are generally stable but that there is more variability overtime in the experience of more severe symptoms.

The aims of this study were to use nationally representative data to examine how a single assessment of broad symptoms of psychological distress is related to measurement of distress over longer periods and to quantify the misclassification arising from using a single assessment to indicate chronic distress. We focus our analyses on those with initially low and high (compared to intermediate) levels of distress: the former is most commonly used as the reference category in epidemiological studies and the latter is associated with the greatest elevation in risk of adverse outcomes.

## Methods

### Sample

This study used data from the Household, Income and Labour Dynamics in Australia (HILDA) Survey, a nationally representative household-based panel study, with annual data collection since 2001 [20]. The sample was selected using a multi-stage probability approach that selected dwellings within Census Collection Districts and households within dwellings; all adults aged 15 years and over within each household were invited to participate. The sample is dynamic; loss occurs when sample members die or are lost to follow-up, and new sample members are added if they join a participating household or when children in participating households turn 15 years of age. The majority of the data are collected with an interviewer-administered Person Questionnaire, however some data relating to factors such as health status and social attitudes are collected with the Self-Completion Questionnaire (SCQ). The K10, assessed with the SCQ, was first included in the Survey in wave 7 and has subsequently been included biennially. Given this, we used data from waves 7, 9, 11, 13 and 15.

At baseline, 7682 households and 13,969 individuals participated in the survey (a 66% household response rate) [21]. Attrition in the HILDA Survey is comparable to other surveys of a similar nature; approximately 90% of participants are reinterviewed at each wave and 85–90% of these participants return the SCQ [22]. Given our focus on individual change in distress over time, we used a balanced panel, limiting our analysis to those who had a valid psychological distress score in waves 7, 9, 11, 13 and 15. Weights, described in detail below, were applied to increase the generalisability of our findings based on the balanced panel. Participants aged < 25 years at wave 7 were also excluded from the analysis. Wave 7 was used as the baseline in this study, and waves 9, 11, 13 and 15 were used to capture a two-, four-, six- and eight-year follow-up period, respectively.

### Measures

Our primary measure was the K10 [10], a tool designed to identify those living in the community with mental disorder, including depression and anxiety, by measuring broad symptoms of psychological distress. Scores on the K10 range from 10 (indicating low levels of distress and low probability of having a disorder) to 50 (severe levels of distress and high probability of having a mental disorder). A range of different cut points are suggested for the K10, and we categorised scores as: low (K10 scores: 10- < 12), mild (12- < 16), moderate (16- < 22) and high (22–50) levels of psychological distress. We also measured age (categorised as: 25- < 45 years, 45- < 65 years and ≥ 65 years) and sex (men and women) at wave 7.

### Statistical approach

As K10 scores are regularly categorised in research and clinical settings, our primary aim was to examine how a single measure of psychological distress is related to measures of distress over time, referred to statistically as ‘agreement’ in categories of distress [23]. To assess agreement, we first described the proportions within initial categories of distress who reported each category of distress at the four follow-up periods. Agreement was defined as being categorised as having the identical category of distress and broader agreement (assessed for low and high distress only) was defined as reporting the same or adjacent category of distress. In order to gauge agreement over the full follow-up period (rather than wave to wave), we also estimated the proportion within each initial category of distress who reported low, low or mild, high, or high or moderate psychological distress never, on 1 occasion or 2–4 (out of 4) occasions over the eight-year period.

Second, we estimated change in continuous K10 scores overall and separately according to baseline category of distress. In order to account for ageing effects and regression to the mean, we used a linear mixed model with a random intercept. The outcome was change in K10 score between baseline and each subsequent wave (representing two-, four-, six- and eight-year change) and we included wave and baseline category of distress in the model, in addition to age group at baseline and sex. Age and sex differences in changes in K10 scores over time were examined with stratified analyses, and by adding interaction terms between baseline category of distress and sex and age group, assessed separately with Wald tests. We also tested a baseline category of distress by wave interaction term into order to assess whether changes over time varied in relation to categories of distress.

In order to make inferences about measurement of psychological distress over time in the general Australian population, it was essential to take the complex survey design into account. The HILDA Survey provides balanced panel responding person weights which account for non-response to the Person Questionnaire, but not non-response to the SCQ. We therefore conducted a logistic regression analysis to predict completion of the SCQ at waves 7, 9, 11, 13 and 15, among the balanced responding person sample, using the same predictors used to derive the longitudinal HILDA person level weights [24]. The inverse of the predicted probabilities of returning the SCQ at each wave were then multiplied by the HILDA provided longitudinal responding person weights to further adjust for SCQ non-response and produce accurate population estimates. Extreme inverse predicted probabilities were truncated at the value that was the median plus six times the interquartile range, set *a priori*, as per previous research [25]. At this stage in the analyses, we also investigated whether psychological distress at wave 7 predicted sample attrition, over and above the factors used to derive weights. A test for trend across K10 categories was performed by including the K10 as an ordinal variable. 95% confidence intervals were estimated using the Taylor Series Linearisation method.

Supplementary analyses present change in K10 scores and categories prior to the final assessment (in wave 15). The HILDA Survey was approved by the University of Melbourne Human Research Ethics Committee.

## Results

In wave 7, there were 12,789 individual respondents from 9464 households, 89% of whom completed the SCQ. Of these, 10,351 respondents aged 25 years and over at wave seven, 2756 (26.6%) were excluded because they did not respond to all four follow-up waves and a further 1456 (14.1%) were excluded because they did not return the SCQ at every follow-up wave used in this study. Among those remaining (*n* = 6139), 170 respondents (2.8%) were excluded because they did not have a valid K10 score at every wave, leaving a final analysis sample of 5969 respondents. Overall, 26.3% of the sample was categorised as having low psychological distress at baseline, 38.9% mild, 19.9% moderate and 14.9% high levels of distress. The weighted characteristics of the study population are presented in Table 1.

**Table 1:** Characteristics of the study population at wave 7 (weighted % and 95 confidence interval)

	Level of psychological distress				Total
	Low	Mild	Moderate	High	
Total	26 (25–28)	39 (37–41)	20 (18–22)	15 (13–17)	100
Sex					
Men	53 (51–56)	48 (46–51)	46 (42–51)	45 (38–51)	49 (47–50)
Women	47 (44–49)	52 (50–54)	54 (49–58)	56 (49–62)	51 (50–53)
Age Group					
25–34	19 (16–22)	23 (20–26)	25 (21–31)	27 (21–34)	23 (21–25)
35–44	21 (19–24)	25 (22–27)	25 (21–29)	26 (20–33)	24 (22–26)
45–54	20 (17–22)	22 (20–25)	24 (20–28)	22 (18–27)	22 (20–24)
55–64	21 (18–25)	17 (16–20)	15 (12–19)	16 (12–20)	18 (16–19)
65–75	14 (12–16)	9 (7–11)	8 (6–11)	6 (4–8)	10 (9–11)
75+	5 (4–7)	4 (3–6)	3 (2–5)	3 (2–6)	4 (3–5)

Percentages are given as column percent with the exception of the total sample, which is given as a row percent

After accounting for factors used to derive weights, compared to those with low distress, the odds of returning the SCQ at every follow-up wave were 0.98 for mild (95% CI: 0.82–1.17), 0.75 for moderate (95% CI:0.62–0.91) and 0.71 for high (95% CI: 0.57–0.87) psychological distress (test for linear trend: *p* < 0.001).

### Agreement in categories of psychological distress

**Fig. 1:**
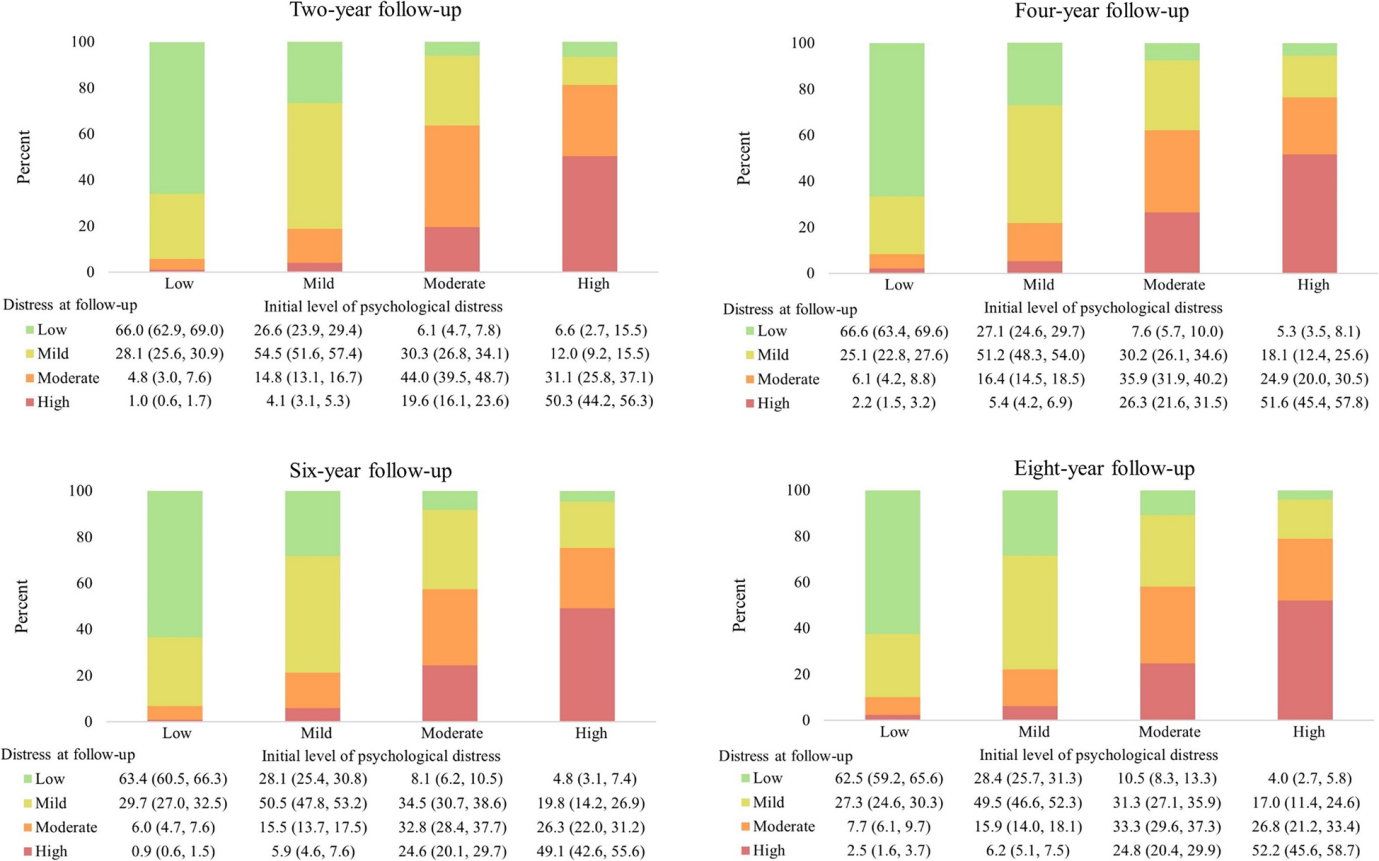
Proportion (and 95% confidence interval) within each initial category of distress with low, mild, moderate and high psychological distress two-, four-, six- and eight-years following initial assessment. Notes: Percentages are given as column percent

The proportions of adults within each baseline category of distress with identical categories of psychological distress (i.e. the same category of psychological distress as at baseline) 2 years following initial assessment was 66.0% for low distress, 54.5% for mild, 44.0% moderate and 50.3% for high distress (Fig. 1). Agreement in low and high distress increased when considering the proportion in the adjacent category: 94.1% of those initially categorised as having low distress had either low or mild distress 2 years later and 81.4% of those who had high levels of distress initially had moderate or high distress 2 years later. Just 5.8% of adults with initially low levels of distress had moderate (4.8%) or high (1.0%) distress 2 years later. Reduction in levels of distress was more common than this, but the overall proportions remained relatively low: 18.6% of adults with high distress had improved scores such that they had low (6.6%) or mild (12.0%) levels of distress 2 years following initial assessment. Agreement in categories of distress did not vary materially when examined over longer follow-up periods. The proportions with identical categories of distress 4 years following baseline assessment were 66.6, 51.2, 35.9 and 51.6% for low, mild, moderate and high distress respectively (Fig. 1). The proportions were similar 8 years after initial assessment: 62.5% for low, 49.5% for mild, 33.3% moderate and 52.2% high. Eight years following the initial assessment, 89.8% of those with low initial levels of distress continued to have low (62.5%) or mild (27.3%) levels of distress and 79.0% of those with initially high levels of distress had moderate (26.8%) or high (52.2%) levels of distress. Eight years following initial assessment, 7.7% of those with low distress initially were categorised as having moderate distress and 2.5% were categorised as having high distress. Similarly, of those with high levels of distress initially, 4.0 and 17.0% had improved scores and were categorised as having low or mild distress, respectively.

Agreement in level of psychological distress was not materially different when assessing retrospective change, that is, change in categories of distress two-, four-, six- and eight- years prior to the final assessment of distress (wave 15) (Supplementary Tables 1 and 2).

**Fig. 2:**
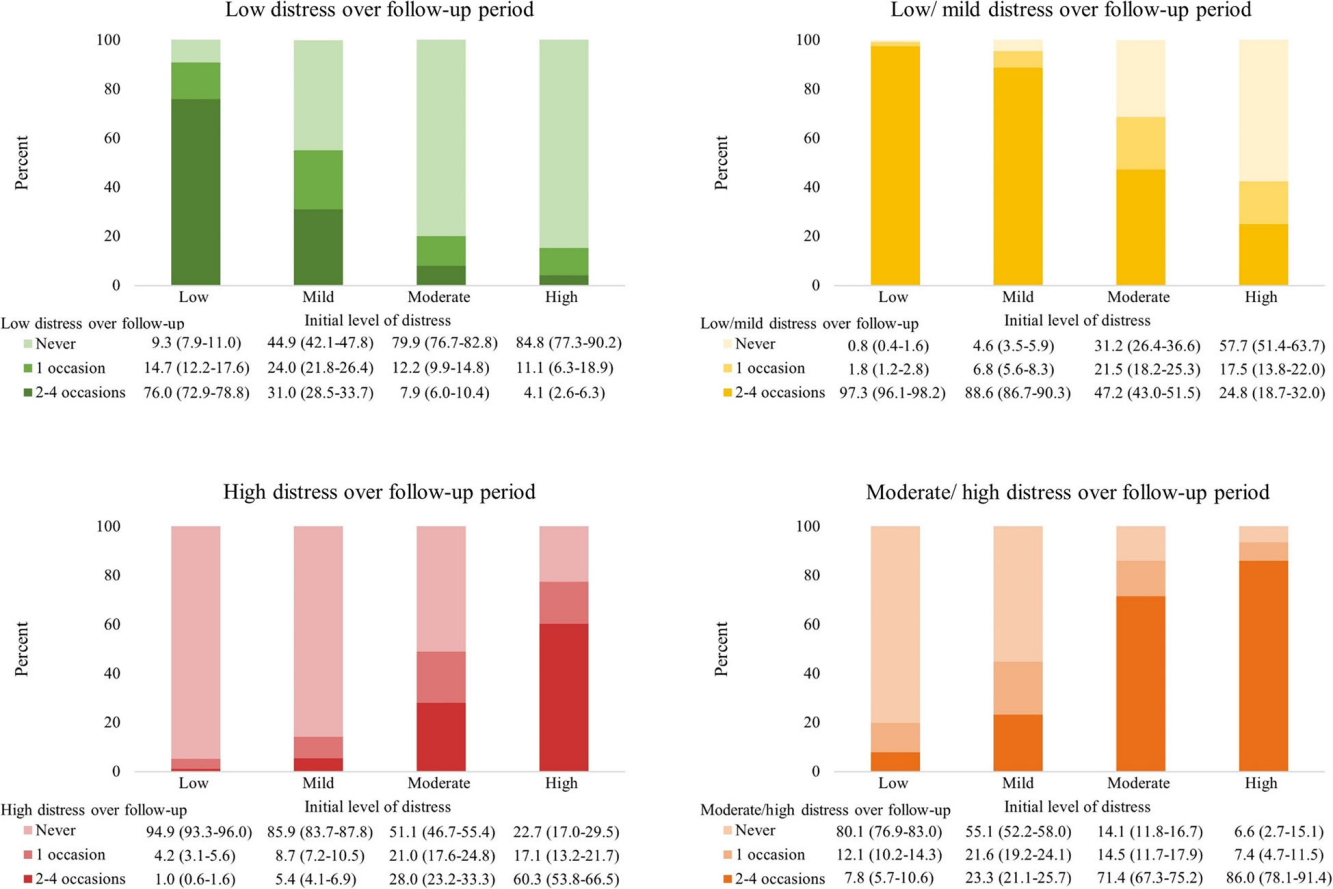
Proportions with low, mild, moderate and high distress initially who report low, low or mild, high and moderate or high never, on 1 occasion or 2–4 occasions over the eight-year follow-up period, according to initial category of distress Notes: Percentages are given as column percent

Over the full eight-year period, of those with initially high distress, 77.3% had high distress on at least one more follow-up occasion, including 60.3% who had high distress on two or more occasions (Fig. 2). However, around one-fifth (22.7%) of those with high distress initially did not have high distress at another point in the follow-up period. The majority (84.8%) of those with initially high distress never had low distress during the follow-up period. Similarly, the overwhelming majority (94.9%) of adults with low initial levels of distress never had high levels of distress. Of those who initially had low levels of distress, more than three-quarters (76.0%) had low levels of distress on most (2–4) follow-up occasions and 9.3% never had low levels of distress again.

### Change in continuous K10 scores

After adjustment for age group and sex, average two-year change in K10 scores was − 0.2 points overall, and 1.1, 0.5, − 0.7 and − 4.9 points for those with low, mild, moderate and high psychological distress, respectively (Table 2). Although wave was a significant predictor in the model (F[1, 389]=19.47, *p* < 0.001), average change in K10 score for each initial category of distress was similar as follow-up time increased: eight years after initial assessment, K10 scores changed on average by 1.5, 0.8, − 0.4 and − 4.6 respectively for individuals with low, mild, moderate and high psychological distress at baseline. There was no evidence that change in K10 scores over time varied by age group, F(15, 375) = 1.47, *p* = 0.115, or sex, F(3, 387) = 1.17, *p* = 0.319 (age- and sex-stratified analyses are provided in Supplementary Tables 3, 4, 5, 6 and 7). There was also little evidence of an interaction between baseline category of distress and wave, F(9, 381) = 1.07, *p* = 0.388.

**Table 2:** Age-sex-adjusted K10 scores at baseline, and average change (and 95% confidence interval) in scores by initial category of distress two-, four-, six- and eight-years following initial assessment, among the Australian population aged 25 years and over

	Initial category of distress				Total
	Low	Mild	Moderate	High	
Average initial score	10.5 (10.5,10.5)	13.3 (13.2,13.4)	18.1 (17.9,18.3)	27.3 (26.7, 27.9)	15.6 (15.5, 15.7)
Two-year change	1.2 (1.1, 1.3)	0.5 (0.4, 0.7)	−0.7 (− 0.9, − 0.5)	−4.9 (− 5.4, − 4.4)	− 0.2 (− 0.3, 0.1)
Four-year change	1.3 (1.2, 1.5)	0.7 (0.5, 0.8)	−0.6 (− 0.8, − 0.3)	− 4.7 (− 5.2, − 4.3)	− 0.1 (− 0.2, 1.0)
Six-year change	1.3 (1.1, 1.4)	0.6 (0.4, 0.8)	−0.6 (− 0.9, − 0.4)	− 4.8 (− 5.3, − 4.3)	− 0.1 (− 0.2, 0.4)
Eight-year change	1.5 (1.4, 1.7)	0.8 (0.7, 1.0)	−0.4 (− 0.6, − 0.1)	− 4.6 (− 5.1, − 4.1)	0.2 (0.0, 0.3)
Total change	1.3 (1.2, 1.4)	0.7 (0.5, 0.8)	−0.6 (− 0.8, − 0.3)	−4.7 (− 5.2, − 4.3)	− 0.0 (− 0.1, 0.1)

Two-, four-, six- and eight-year change scores were estimated using linear mixed model with a random intercept, and are adjusted for age group, sex at baseline

## Discussion

Levels of psychological distress were broadly stable over time in this nationally representative survey. Approximately 90% of people who initially had low levels of distress continued to report low or mild levels of distress over 8 years and around 75% with initially high levels of distress remained moderately or highly distressed. Furthermore, around half of all adults had identical categories of distress every 2 years over the eight-year follow-up period. Over the full eight-year follow-up, more than 75% of people with high distress initially were highly distressed on at least one more occasion, including more than half (60.3%) who reported a pattern consistent with chronically high symptoms of distress. Change in continuous scores was on average less than half a point at each follow-up period, but largest (almost five points) for those with high levels of distress initially.

This is the first study to use a nationally representative sample to examine the extent to which single measures of distress can be used to indicate longer-term experience of distress. Our results are broadly consistent with a number of previous studies reporting broad stability in measures of distress over time. Using structural equation models, one study reported that psychological distress was relatively stable over an up to 10 year period, with no discernible difference in the estimates using longer compared to shorter follow-up periods [18]. Similarly, a number of studies describing trajectories in depressive symptoms have found that more than 80% of mid-and older-age individuals in the general community report stable depressive symptoms over a 10-year period, with the majority of participants continuing to report no, low, or only moderate depressive symptoms [26–28]. Previous research has also demonstrated that depressive symptoms are relatively stable in a number of subpopulations, including women transitioning from pre-pregnancy to the perinatal period [29], post-natal mothers [30], young adults with type 1 diabetes [31] and mothers with children with autism spectrum disorder [32]. Our study builds on these findings by examining change in relation to initial category of distress and providing estimates of the amount of misclassification likely to be present in studies which use a single assessment to indicate chronic symptoms of psychological distress.

In this study it is not possible to ascertain the extent to which the high degree of broad agreement in measurement of high distress reflects chronic mental disorders (e.g. dysthymia), a general propensity to experience high psychological distress, multiple episodes of high distress, or chronic external stressors. However, it is likely that all four at least partly explain our findings [33]. Stable, trait-based personality factors have been shown to play a key role in determining levels of distress, accounting for between half and two-thirds of the difference between individuals in levels of distress in some studies, with the remainder attributed to external factors [34–36]. While levels of psychological distress are known to fluctuate in reaction to stressful life events, many key stressful life event such as physical illness, financial stressors or grief are likely to be experienced long term. Furthermore, coping strategies, which can moderate the association between external stressors and psychological distress, are also generally stable over time [37].

Our findings indicate that, in addition to identifying individuals with a high probability of a common mental disorder at the time of screening, the K10 can be used to identify those with long-term (i.e. up to at least 8-years) vulnerability to common mental disorders. That is, while the presence of a diagnosable mood and or anxiety disorder is often episodic, the broader experience of symptoms of distress is relatively consistent over time. Thus, for the majority of individuals who participate in cohort studies, single assessments of distress are likely to provide a good indication of likely caseness or subsyndromal symptoms experienced over longer follow-up periods. Though there will be some fluctuation, the vast majority of those with low distress will continue to report low distress (resulting in a relatively stable reference group) and most individuals with high distress will experience it for more than a single short period. While repeated measures are the gold standard to identify those who experience reoccurring or chronic symptoms of distress, they are also onerous for participants, expensive and often impractical. Given broad agreement in K10 scores over time, it is feasible to use single assessments of distress as a proxy for the experience of chronic distress in epidemiological research. We estimate that approximately one-fifth of those with high distress will be incorrectly classified as having long-term distress when using this method. Future studies which use single assessments to indicate longer-term distress can use the estimates presented in this study to conduct bias analyses to quantify the direction and magnitude of the bias resulting from distress misclassification [38]. However, given that average change in scores for those with high psychological distress was less than five points on average, it is likely that those who are incorrectly classified continue to have relatively high levels of distress, suggesting minimal impacts on estimates of association.

Our finding that, for many people, levels of psychological distress are broadly stable over relatively long follow-up periods helps to explain the associations between common mental disorders and outcomes, such as chronic physical disease. While the reasons that poor mental health is associated with physical illness remain unclear, including the extent to which poor mental health results in poor physical health or vice versa, multiple pathways have been proposed [39]. Regardless of the underlying mechanisms, the strength of association between common mental disorders and physical health is likely to be related to the amount of time exposed to symptoms of distress [40]. Given that we show that a single assessment of distress is a reasonable proxy for the experience of longer-term distress, the risk of poor physical outcomes is likely to reflect, at least in part, that the majority of those with high levels of distress experience symptoms for substantial periods of time.

### Strengths and limitations

We used nationally representative data to describe change in distress over multiple time points, spanning an eight-year period. Rather than providing a summary measure, we stratified our analyses in relation to initial level of distress, which allowed a more detailed analysis of change across categories of distress. Levels of distress were assessed at two-year intervals, and given that most common mental disorders are episodic, it is likely there was change in distress during the time between assessments and that we missed respondents who reported high levels of distress outside the time window of reporting. Certain sociodemographic groups, particularly immigrants, are underrepresented in the HILDA Survey. Weights, benchmarked to the Australian population, have been used to correct for attrition in the Survey but generalisations to the broader population should be made with this in mind. Furthermore, K10 scores predicted attrition and non-response to the SCQ over the study period, over and above the factors used to estimate sample weights. This, combined with the survivor effects in the balanced panel sample, likely resulted in an underestimate of true agreement in symptoms of distress because we have underestimated the proportion of the population who experience higher distress (and this group reported less agreement in categories of distress over time) as well as the proportion with consistently high distress. Nonetheless, we demonstrated broad continuity in high psychological distress amongst those in our sample.

## Conclusion

Single assessments of psychological distress are regularly used in a range of studies as indicators of chronic mental health problems and used to predict outcomes over long follow-up periods, years after initial assessment of distress. This study has demonstrated that while there is a degree of change, the experience of symptoms of distress is broadly stable, with the majority of individuals continuing to report the same or a similar level of distress over an eight-year period. Given the broad agreement in measures of distress over time, in the absence of repeated measures, single assessments of distress can be used as a proxy for chronic symptoms of distress and bias analyses can be used to quantify the effect of misclassification.

## Supplementary information

**Additional file 1: Supplementary Tables.** Description of data: Supplementary tables describing retrospective change in K10 scores in relation to category of distress at the final assessment. Supplementary tables describing K10 scores at baseline, and average change in scores by initial category of distress two-, four-, six- and eight-years following initial assessment stratified by age and sex.

## Data Availability

Information relating to access to the Household, Income and Labour Dynamics in Australia (HILDA) Survey can be found here: https://melbourneinstitute.unimelb.edu.au/hilda/for-data-users

## Abbreviations

HILDA: Household, Income and Labour Dynamics in Australia
IQR: Interquartile Range
K10: Kessler 10
SCQ: Self Completion Questionnaire
