# Supplementary material for: Identifying long-term psychological distress from single measures: evidence from a nationally representative longitudinal survey of the Australian population
Source: BMC Med Res Methodol. 2020 Mar 5;20:55. doi: 10.1186/s12874-020-00938-8 (PMC7059354; doi:10.1186/s12874-020-00938-8)
Supplement: Supplementary file 1 — Additional file 1: Supplementary Tables. Description of data: Supplementary tables describing retrospective change in K10 scores in relation to category of distress at the final assessment. Supplementary tables describing K10 scores at baseline, and average change in scores by initial category of distress two-, four-, six- and eight-years following initial assessment stratified by age and sex. [file 12874_2020_938_MOESM1_ESM.docx]

# List of supplementary tables

[List of supplementary tables 1](#_Toc32485231)

[Table S1. Proportion (and 95% confidence interval) within each initial category of distress at the final assessment (wave 15) with low, mild, moderate and high psychological distress two-, four-, six- and eight-years prior to the final assessment, among the Australian population aged 25 years and over. 2](#_Toc32485232)

[Table S2. Proportions with low, mild, moderate and high distress never, on 1 occasion or 3-4 occasions over the eight-year period before the final assessment, according to final category of distress, among the Australian population aged 25 years and over. 3](#_Toc32485233)

[Table 3. Age-adjusted K10 scores at baseline, and average change (and 95% confidence interval) in scores by initial category of distress two-, four-, six- and eight-years following initial assessment, among Australian men aged 25 years and over. 4](#_Toc32485234)

[Table 4. Age-adjusted K10 scores at baseline, and average change (and 95% confidence interval) in scores by initial category of distress two-, four-, six- and eight-years following initial assessment, among Australian women aged 25 years and over. 4](#_Toc32485235)

[Table 5. Age-adjusted K10 scores at baseline, and average change (and 95% confidence interval) in scores by initial category of distress two-, four-, six- and eight-years following initial assessment, among the Australian population women aged 25-44 years. 5](#_Toc32485236)

[Table 6. Age-adjusted K10 scores at baseline, and average change (and 95% confidence interval) in scores by initial category of distress two-, four-, six- and eight-years following initial assessment, among the Australian population women aged 45-64 years. 5](#_Toc32485237)

[Table 7. Age-adjusted K10 scores at baseline, and average change (and 95% confidence interval) in scores by initial category of distress two-, four-, six- and eight-years following initial assessment, among the Australian population women aged 65 years and over. 5](#_Toc32485238)

## Table S1. Proportion (and 95% confidence interval) within each initial category of distress at the final assessment (wave 15) with low, mild, moderate and high psychological distress two-, four-, six- and eight-years prior to the final assessment, among the Australian population aged 25 years and over.

|  | Final category of distress | | | |
| --- | --- | --- | --- | --- |
|  | Low  % (95%CI) | Mild  % (95%CI) | Moderate  % (95%CI) | High  % (95%CI) |
| **Distress two-years prior** |  |  |  |  |
| Low | 66.0 (63.1-68.8) | 23.4 (20.9-26.0) | 6.1 (4.3-8.4) | 4.0 (2.6-6.2) |
| Mild | 29.0 (26.3-31.8) | 56.8 (53.5-60.1) | 34.5 (30.4-38.8) | 12.8 (10.1-16.0) |
| Moderate | 3.6 (2.7-4.8) | 15.5 (13.5-17.7) | 38.9 (35.2-42.8) | 26.5 (21.9-31.7) |
| High | 1.4 (0.6-3.3) | 4.3 (2.8-6.5) | 20.5 (16.9-24.6) | 56.7 (50.5-62.7) |
| **Distress four-years prior** |  |  |  |  |
| Low | 63.4 (60.5-66.1) | 24.9 (22.4-27.5) | 9.8 (7.6-12.7) | 4.0 (2.5-6.1) |
| Mild | 28.9 (26.1-31.8) | 52.0 (48.8-55.2) | 30.9 (27.4-34.7) | 15.0 (11.5-19.1) |
| Moderate | 6.4 (4.3-9.5) | 17.9 (15.6-20.3) | 36.7 (32.3-41.3) | 23.6 (19.6-28.1) |
| High | 1.4 (0.8-2.2) | 5.2 (4.0-6.8) | 22.6 (18.6-27.1) | 57.5 (51.9-63.0) |
| **Distress six-years prior** |  |  |  |  |
| Low | 59.3 (56.0-62.4) | 27.5 (24.4-30.8) | 9.8 (7.7-12.4) | 3.2 (2.0-5.1) |
| Mild | 32.8 (30.0-35.8) | 50.6 (47.4-53.8) | 33.0 (29.0-37.4) | 15.9 (12.9-19.3) |
| Moderate | 6.4 (4.5-8.9) | 16.5 (14.3-18.9) | 37.3 (33.2-41.6) | 36.0 (30.0-42.5) |
| High | 1.6 (1.0-2.4) | 5.5 (3.8-7.8) | 19.9 (16.6-23.6) | 44.9 (39.4-50.6) |
| **Distress eight-years prior** |  |  |  |  |
| Low | 54.5 (51.3-57.6) | 20.4 (18.2-22.9) | 10.8 (8.5-13.5) | 4.1 (2.7-6.2) |
| Mild | 36.6 (33.7-39.7) | 54.7 (51.4-58.0) | 32.9 (28.9-37.0) | 15.2 (12.4-18.6) |
| Moderate | 6.9 (5.4-8.8) | 17.7 (15.5-20.1) | 35.2 (30.8-39.8) | 31.3 (25.5-37.8) |
| High | 2.0 (1.3-2.9) | 7.2 (4.7-10.9) | 21.2 (16.7-26.6) | 49.3 (43.5-55.2) |

Notes: Percentages given are column percent.

## Table S2. Proportions with low, mild, moderate and high distress never, on 1 occasion or 3-4 occasions over the eight-year period before the final assessment, according to final category of distress, among the Australian population aged 25 years and over.

|  | Final category of distress | | | |
| --- | --- | --- | --- | --- |
|  | Low  % (95%CI) | Mild  % (95%CI) | Moderate  % (95%CI) | High  % (95%CI) |
| **Low distress in the eight-years prior** | |  |  |  |
| Never | 12.1 (10.3-14.2) | 49.6 (46.2-53.0) | 76.9 (73.4-80.1) | 89.2 (86.1-91.6) |
| 1 occasion | 16.4 (14.1-19.0) | 23.7 (20.8-26.9) | 13.8 (11.4-16.6) | 8.0 (5.9-10.8) |
| 2-4 occasion | 71.5 (68.7-74.2) | 26.7 (24.2-29.4) | 9.3 (7.1-12.0) | 2.8 (1.7-4.7) |
| **Low/mild distress in the eight-years prior** | |  |  |  |
| Never | 0.7 (0.3-1.4) | 4.9 (3.5-6.9) | 27.2 (23.2-31.6) | 61.4 (55.8-66.7) |
| 1 occasion | 1.3 (0.8-2.2) | 7.6 (6.2-9.3) | 21.9 (18.9-25.2) | 18.0 (14.4-22.2) |
| 2-4 occasion | 98.0 (97-98.7) | 87.5 (85.0-89.6) | 50.9 (46.1-55.8) | 20.6 (17.1-24.7) |
| **High distress in the eight-years prior** | |  |  |  |
| Never | 95.2 (93.5-96.5) | 84.8 (81.2-87.8) | 52.8 (47.8-57.7) | 18.1 (14.6-22.2) |
| 1 occasion | 3.8 (2.7-5.4) | 10.0 (7.5-13.2) | 23.9 (20.8-27.3) | 17.2 (13.9-21.1) |
| 2-4 occasion | 1.0 (0.5-1.9) | 5.2 (3.5-7.7) | 23.3 (19.1-28.1) | 64.7 (59.3-69.7) |
| **Moderate/high distress in the eight-years prior** | | |  |  |
| Never | 79.9 (76.5-82.9) | 52.3 (48.9-55.7) | 13.4 (10.9-16.3) | 4.2 (2.7-6.4) |
| 1 occasion | 13.2 (11.3-15.4) | 23.2 (20.2-26.4) | 17.2 (14.4-20.4) | 6.5 (4.9-8.7) |
| 2-4 occasion | 6.9 (5.0-9.3) | 24.5 (21.9-27.4) | 69.4 (65.2-73.3) | 89.3 (86.4-91.6) |

Notes: Percentages given are column percent.

## Table 3. Age-adjusted K10 scores at baseline, and average change (and 95% confidence interval) in scores by initial category of distress two-, four-, six- and eight-years following initial assessment, among Australian men aged 25 years and over.

|  | Initial category of distress | | | | Total |
| --- | --- | --- | --- | --- | --- |
|  | Low | Mild | Moderate | High |  |
| Average initial score | 10.5 (10.4-10.5) | 13.3 (13.2-13.4) | 18.0 (17.8-18.2) | 26.6 (25.7-27.6) | 15.2 (15.0-15.3) |
| Two-year change | 1.0 (0.8, 1.2) | 0.3 (0.1, 0.5) | -1.0 (-1.4, -0.6) | -4.6 (-5.3, -3.9) | -0.3 (-0.4, -0.1) |
| Four-year change | 1.3 (1.1, 1.5) | 0.6 (0.4, 0.8) | -0.7 (-1.1, -0.4) | -4.4 (-5.1, -3.7) | 0.0 (-0.2, 0.2) |
| Six-year change | 1.2 (1.0, 1.3) | 0.5 (0.2, 0.7) | -0.9 (-1.2, -0.5) | -4.5 (-5.2, -3.8) | -0.1 (-0.3, 0.1) |
| Eight-year change | 1.5 (1.3, 1.7) | 0.8 (0.6, 1.0) | -0.5 (-0.9, -0.2) | -4.1 (-4.9, -3.4) | 0.3 (0.1, 0.4) |
| Total change | 1.2 (1.1, 1.4) | 0.5 (0.4, 0.7) | -0.8 (-1.1, -0.4) | -4.4 (-5.1, -3.7) | 0.0 (-0.2, 0.1) |

Notes: Two-, four-, six- and eight-year change scores were estimated using linear mixed model with a random intercept, and are adjusted for age group at baseline.

## Table 4. Age-adjusted K10 scores at baseline, and average change (and 95% confidence interval) in scores by initial category of distress two-, four-, six- and eight-years following initial assessment, among Australian women aged 25 years and over.

|  | Initial category of distress | | | | Total |
| --- | --- | --- | --- | --- | --- |
|  | Low | Mild | Moderate | High |  |
| Average initial score | 10.5 (10.5-10.6) | 13.3 (13.2-13.3) | 18.2 (18-18.5) | 27.9 (27.2-28.5) | 16.0 (15.9-16.1) |
| Two-year change | 1.3 (1.2, 1.5) | 0.7 (0.5, 0.9) | -0.5 (-0.8, -0.1) | -5.0 (-5.7, -4.4) | -0.1 (-0.3, 0.0) |
| Four-year change | 1.4 (1.2, 1.6) | 0.7 (0.5, 0.9) | -0.4 (-0.8, -0.1) | -5.0 (-5.6, -4.4) | -0.1 (-0.3, 0.1) |
| Six-year change | 1.4 (1.2, 1.6) | 0.7 (0.5, 0.9) | -0.4 (-0.8, -0.1) | -5.0 (-5.7, -4.4) | -0.1 (-0.3, 0.1) |
| Eight-year change | 1.5 (1.3, 1.7) | 0.9 (0.7, 1.1) | -0.3 (-0.7, 0.1) | -4.9 (-5.5, -4.2) | 0.1 (-0.1, 0.3) |
| Total change | 1.4 (1.3, 1.5) | 0.7 (0.6, 0.9) | -0.4 (-0.8, -0.1) | -5.0 (-5.6, -4.3) | -0.1 (-0.2, 0.1) |

Notes: Two-, four-, six- and eight-year change scores were estimated using linear mixed model with a random intercept, and are adjusted for age group at baseline.

## Table 5. Age-and sex-adjusted K10 scores at baseline, and average change (and 95% confidence interval) in scores by initial category of distress two-, four-, six- and eight-years following initial assessment, among the Australian population women aged 25-44 years.

|  | Initial category of distress | | | | Total |
| --- | --- | --- | --- | --- | --- |
|  | Low | Mild | Moderate | High |  |
| Average initial score | 10.5 (10.5-10.6) | 13.3 (13.2-13.4) | 18.2 (17.9-18.5) | 27.3 (26.5-28.2) | 16.1 (15.9-16.3) |
| Two-year change | 1.5 (1.3, 1.8) | 0.7 (0.4, 0.9) | -0.6 (-1.1, -0.1) | -5.6 (-6.4, -4.8) | -0.2 (-0.4, -0.1) |
| Four-year change | 1.6 (1.3, 1.9) | 0.7 (0.5, 1.0) | -0.5 (-1.0, 0.0) | -5.5 (-6.4, -4.7) | -0.2 (-0.4, 0.1) |
| Six-year change | 1.6 (1.3, 1.9) | 0.7 (0.4, 1.0) | -0.5 (-1.0, 0.0) | -5.5 (-6.4, -4.7) | -0.2 (-0.4, 0.1) |
| Eight-year change | 2.1 (1.8, 2.4) | 1.3 (0.9, 1.6) | 0.0 (-0.5, 0.6) | -5.0 (-5.9, -4.1) | 0.3 (0.0, 0.7) |
| Total change | 1.7 (1.4, 1.9) | 0.8 (0.6, 1.1) | -0.4 (-0.9, 0.0) | -5.5 (-6.3, -4.6) | -0.1 (-0.3, 0.1) |

Notes: Two-, four-, six- and eight-year change scores were estimated using linear mixed model with a random intercept, and are adjusted for sex.

## Table 6. Age-and sex-adjusted K10 scores at baseline, and average change (and 95% confidence interval) in scores by initial category of distress two-, four-, six- and eight-years following initial assessment, among the Australian population women aged 45-64 years.

|  | Initial category of distress | | | | Total |
| --- | --- | --- | --- | --- | --- |
|  | Low | Mild | Moderate | High |  |
| Average initial score | 10.5 (10.4-10.5) | 13.3 (13.2-13.4) | 18.1 (17.8-18.4) | 27.5 (26.7-28.3) | 15.5 (15.4-15.6) |
| Two-year change | 1.3 (1.1, 1.4) | 0.7 (0.5, 0.9) | -0.7 (-1.0, -0.4) | -4.2 (-4.8, -3.6) | -0.1 (-0.2, 0.1) |
| Four-year change | 1.4 (1.2, 1.6) | 0.8 (0.6, 1.0) | -0.6 (-0.9, -0.2) | -4.1 (-4.7, -3.5) | 0.1 (-0.1, 0.2) |
| Six-year change | 1.3 (1.1, 1.5) | 0.7 (0.5, 1.0) | -0.6 (-1.0, -0.3) | -4.1 (-4.8, -3.5) | 0.0 (-0.2, 0.2) |
| Eight-year change | 1.3 (1.1, 1.5) | 0.7 (0.5, 1.0) | -0.7 (-1.0, -0.3) | -4.2 (-4.8, -3.5) | 0.0 (-0.2, 0.2) |
| Total change | 1.3 (1.2, 1.5) | 0.7 (0.5, 0.9) | -0.7 (-1.0, -0.3) | -4.2 (-4.8, -3.5) | 0.0 (-0.2, 0.1) |

Notes: Two-, four-, six- and eight-year change scores were estimated using linear mixed model with a random intercept, and are adjusted for sex.

## Table 7. Age-and sex-adjusted K10 scores at baseline, and average change (and 95% confidence interval) in scores by initial category of distress two-, four-, six- and eight-years following initial assessment, among the Australian population women aged 65 years and over.

|  | Initial category of distress | | | | Total |
| --- | --- | --- | --- | --- | --- |
|  | Low | Mild | Moderate | High |  |
| Average initial score | 10.5 (10.4-10.5) | 13.2 (13-13.4) | 17.9 (17.6-18.2) | 26.5 (25.2-27.9) | 14.2 (14.1-14.4) |
| Two-year change | 0.6 (0.4, 0.7) | 0.0 (-0.2, 0.3) | -1.0 (-1.5, -0.6) | -5.1 (-6.0, -4.2) | -0.4 (-0.6, -0.2) |
| Four-year change | 0.8 (0.7, 1.0) | 0.3 (0.1, 0.5) | -0.8 (-1.2, -0.3) | -4.9 (-5.7, -4.0) | -0.1 (-0.3, 0.0) |
| Six-year change | 0.8 (0.6, 0.9) | 0.2 (0.0, 0.5) | -0.8 (-1.3, -0.4) | -4.9 (-5.8, -4.0) | -0.2 (-0.4, 0.0) |
| Eight-year change | 1.3 (1.1, 1.5) | 0.7 (0.5, 1.0) | -0.3 (-0.8, 0.1) | -4.4 (-5.3, -3.5) | 0.3 (0.1, 0.5) |
| Total change | 0.9 (0.8, 1.0) | 0.4 (0.2, 0.6) | -0.7 (-1.1, -0.3) | -4.8 (-5.7, -3.9) | 0.0 (-0.2, 0.1) |

Notes: Two-, four-, six- and eight-year change scores were estimated using linear mixed model with a random intercept, and are adjusted for sex.
